# Decreased low-density lipoprotein receptor-related protein 1 expression in pro-inflammatory monocytes is associated with subclinical atherosclerosis

**DOI:** 10.3389/fcvm.2022.949778

**Published:** 2022-07-26

**Authors:** Ricardo A. Albertini, Juan C. Nicolas, Virginia Actis Dato, Darío G. Ferrer, María E. Tinti, Raúl H. Capra, Gustavo A. Chiabrando

**Affiliations:** ^1^Servicio de Clínica Médica, Hospital Privado Universitario de Córdoba, Instituto Universitario de Ciencias Biomédicas Córdoba, Córdoba, Argentina; ^2^Facultad de Ciencias Químicas, Universidad Católica de Córdoba, Córdoba, Argentina; ^3^Departamento de Bioquímica Clínica, Facultad de Ciencias Químicas, Centro de Investigaciones en Bioquímica Clínica e Inmunología (CIBICI) Consejo Nacional de Investigaciones Cientificas y Tecnicas (CONICET), Universidad Nacional de Córdoba, Córdoba, Argentina; ^4^Servicio de Laboratorios, Hospital Privado Universitario de Córdoba, Instituto Universitario de Ciencias Biomédicas Córdoba, Córdoba, Argentina; ^5^Servicio de Diagnóstico por Imágenes, Hospital Privado Universitario de Córdoba, Instituto Universitario de Ciencias Biomédicas Córdoba, Córdoba, Argentina; ^6^Centro de Investigación en Medicina Traslacional Severo Amuchástegui (CIMETSA), Instituto Universitario de Ciencias Biomédicas Córdoba, Córdoba, Argentina

**Keywords:** cardiovascular, cytokines, inflammation, lipids, lipoproteins, receptors

## Abstract

Subclinical atherosclerosis (SCA) occurs in asymptomatic individuals. Blood peripheral monocytes are involved in the development of atherosclerosis. Circulating monocytes acquire pro-inflammatory profiles, and they are involved in the early stages of atherosclerosis development. Low-density lipoprotein Receptor-related Protein 1 (LRP1) is expressed in monocytes, mainly in classical and intermediate subsets. Although LRP1 is highly expressed in macrophages and vascular smooth muscle cells (VSMCs) in atherosclerotic plaque formation, its expression in circulating monocytes has not been studied in SCA. The aim of this study was to characterize the LRP1 expression level in circulating monocytes of individuals with SCA and compared with individuals with low (LR) and intermediate (IR) risk of cardiovascular diseases, both without evidence of atherosclerotic lesions in carotid and coronary arteries. LRP1 and additional markers (CD11b, CD11c, and CD36) at cell surface of monocytes were analyzed by flow cytometry assays, whereas *LRP1* and pro-inflammatory factors gene expressions were measured in isolated monocytes by quantitative RT-PCRs. Both LRP1 protein and *LRP1* mRNA were significantly reduced in monocytes in SCA and IR respect to LR. Conversely, CD36, CD11b, and CD11c monocytic markers showed no significant changes between the different study groups. Finally, increased gene expressions of *TNF-*α and *IL-1*β were detected in monocytes of SCA, which were associated with decreased LRP1 expression at the cell surface in total monocytes. In summary, we propose that the decreased LRP1 expression at cell surface in total monocytes with pro-inflammatory profile is associated with the development of atherosclerosis in asymptomatic individuals.

## Introduction

The detection by imaging methods of atherosclerotic plaques in arteries of asymptomatic individuals is termed subclinical atherosclerosis (SCA) (1, 2). SCA represents the majority of cases among younger adults, who can suffer cardiovascular events despite being considered as low CVD risk score (1, 3). Thus, SAC underlies most cardiovascular events, and its early detection can improve risk stratification.

Peripheral blood monocytes play a central role during the development and progression of atherosclerosis (4). Monocytes are main cellular component of innate immunity, which can acquire a pro-inflammatory phenotype after certain stimulus, not only with microbial products but also endogenous atherogenic stimulus that are produced by risk factors associated with obesity, type 2 Diabetes Mellitus, and dyslipidemia (5). This persistent activation of circulating monocytes is characterized by an increased gene expression of pro-inflammatory cytokines and growth factors, such as TNF-α, IL-1β, and CCL2 (6, 7). However, this pro-inflammatory profile of monocytes has not been systematically evaluated in individuals with SCA.

Low-density lipoprotein Receptor-related Protein 1 (LRP1) is a cell surface glycoprotein composed by two subunits: A large subunit of 515 kDa (LRP1-α), containing the extracellular binding domains, and a subunit of 85 kDa (LRP1-β) comprising the membrane spanning and cytoplasmic domain (8). Although LRP1 is considered an endocytic receptor, it has been shown its ability to promote intracellular signaling regulating proliferation, migration, and differentiation of different cell types, including macrophages and vascular smooth muscle cells (VSMCs) (9). It has been demonstrated that LRP1 is involved in the atherosclerosis development and atherogenic plaque formation (10, 11). In macrophages, LRP1 is highly expressed and regulates cellular proliferation and migration (12, 13) and foam cell formation through the binding and internalization of aggregated LDL (aggLDL) (14). In addition, LRP1 is involved in the suppression of the Toll-like receptor (TLR)-induced inflammation in macrophages (15) and in consequence LRP1 deficiency is associated with increased expression of pro-inflammatory mediators, included TNF-α and IL-6, in this type of cells (16). LRP1 is expressed in human monocytes (17) but its expression level in association with the pro-inflammatory profile in these circulating cells have not been established yet in SCA individuals.

Thus, the aim of this study was to analyze whether the LRP1 expression level in circulating monocytes from asymptomatic individuals with atherosclerosis is associated with pro-inflammatory phenotypes of these cells.

## Materials and methods

### Study design

Descriptive and analytical observational cross-sectional study was performed on a population sample of asymptomatic adults (20–59 years old) voluntarily enrolled at the Clinical Medicine Service of the Hospital Privado Universitario de Cordoba (HPUC) as previously was published (2). Briefly, from a total of 254 enrolled, 27 were excluded by exclusion criteria (CVD antecedents, diabetes mellitus, lipid-lowering drug treatment and pregnant women), 82 individuals were classified as SCA group, 21 presented low-risk factors of CVD (LR group) and 124 individuals were classified as intermediate-risk factors of CVD (IR group). Criteria for LR, IR, and SCA groups are defined in Supplementary Methods.

### Carotid ultrasound study

The CU study was performed using Accuvix V10, Samsung Medison Co., Ltd. (Seoul, Korea) with a linear transduction of variable frequency from 5 to 13 MHz as was previously described (2) and detailed in Supplementary Methods.

### Coronary artery calcium score determination by cardiac computed tomography

Cardiac computed tomography (CT) scans were performed using a Toshiba Aquilion helicoidal multidetector tomograph manufactured by Toshiba America Medical Systems, Inc. (Tustin, CA), which has 16 detectors in line as was previously described (2) and detailed in Supplementary Methods.

### Laboratory measurements

Fresh whole blood samples were drawn into collection tubes for laboratory tests that included white cell and monocyte measurement, plasma glucose, total cholesterol, triglycerides, low-density lipoprotein cholesterol (LDLc), high-density lipoprotein cholesterol (HDLc), lipoprotein-(a) [Lp(a)], creatinine and high-sensitivity C-reactive protein (hs-CRP). Non-HDLc was calculated from (total cholesterol - HDLc concentrations).

### Flow cytometry analysis

Samples were prepared within 30 min after drawn. About 50 μl of whole blood was added to a 5-ml polystyrene round-bottomed tube (No. 352008; BD Biosciences), and 1 ml of each antigen-specific fluorochrome-labeled antibody was added (antibody dilutions are indicated in Supplementary Table 1 and all isotype controls were purchased from their respective manufacturers). The sample was then incubated for 20 min at 4°C in the dark. Lysis of erythrocytes was performed using a lysing buffer (No. 555899; BD Pharm Lyse) for 15 min. To ensure maximum viability, stained cells were analyzed promptly.

LRP1 in total monocytes and monocyte subsets was measured by flow cytometry using the gating strategy previously described (17) and detailed in Supplementary Methods. For measurement of CD36, CD11b, and CD11c in total monocytes and monocyte subsets by flow cytometry each antigen-specific PerCP-Cy5.5-labeled antibody (dilution indicated in Supplementary Table 1) was added in separate tubes for its corresponding monocyte marker. Next, the MFI for each marker in monocyte subsets was determined from cells gated on CD14 vs. CD16 plot, whereas for total monocytes was defined form the cell distribution pattern obtained in the SSC vs. LRP1-positive plot (Supplementary Figure 1). The total variation yield by the measurement of LRP1, CD36, CD11b, and CD11c in total monocytes and monocyte subsets, calculated from the analytical (*a*CV%) and biological (*b*CV%) variation as [(*a*CV%^2^ + *b*CV%^2^)^1/2^] (18), was not higher than 20% according as was previously described (17).

### Total monocyte isolation by fluorescent activated cell-sorting

For the total monocytes isolation by fluorescent activated cell-sorting (FACS) we employed the gating strategy previously described (17) and schematized in Supplementary Figure 1. Briefly, 6 ml of whole blood were used and the procedure of sample processing for flow cytometry was repeated maintaining the proportion of reagents relative to whole blood volume as was indicated for flow cytometry assays (Supplementary Methods and Supplementary Table 1). The cell sorting was performed with a BD FACS Aria TMIIu cell sorter calibrated with BD Cytometer Setup Tracking Beads (No. 641319, BD Biosciences) and Accudrop Beads (No. 345248, BD Biosciences). BD FACS Diva TM software (version 6.1.2) was used to acquire and analyze the data.

### Quantitative reverse transcriptase-PCR assays

From isolated total monocytes the transcript products of LRP1 and pro-inflammatory factors were analyzed by quantitative reverse transcriptase-PCR assays (qPCR) as is detailed in Supplementary Methods. Sequences of specific primers for *LRP1*, *TNF-*α, *IL-1*β, *CCL2*, *CCR2* and *GAPDH* are indicated in Supplementary Table 2.

### Statistics

Data were presented as mean ± standard deviation (SD) or median and interquartile range (IQR) for continuous variables and frequencies (percentages) for categorical variables. The normality of the data was analyzed using the Kolmogorov–Smirnov test. Parameters with non-normal distribution were log-transformed to achieve normal distribution and to apply statistical parametric analysis, but the original data were used in different visual representations. For statistical analysis, the parametric paired *t*-test and ordinary one-way ANOVA were used. For certain parameters, mean values were adjusted with statistic ANCOVA models. For binary comparison between parameters, Pearson correlation analysis was applied. The statistical software GraphPad Prism 6.2 (San Diego, CA) was used for all statistical analyses. Differences were considered statistically significant when *p* < 0.05.

## Results

To analyze the cell surface LRP1 expression in circulating monocytes at early stages of atherosclerosis we enrolled asymptomatic individuals with SCA criteria (SCA group; *n* = 82) and compared to individuals with low (LR group; *n* = 21) and intermediate (IR group; *n* = 124) risk factors of CVD. Table 1 shows clinical and biochemical parameters of the three study groups (Study I). In the SCA group, the presence of carotid atherosclerotic plaque (CAP) was combined with coronary artery calcium (CAC) scores > 1.0 in 17% of females and 66% of males, which are in agreement with previous studies published (2). From comparison of lipid parameters only total cholesterol, LDLc and non-HDLc shown significant difference between three groups, whereas triglycerides shown significant difference between LR and SCA and between IR and SCA, but not between LR and IR. Then, HDLc shown significant difference between LR and IR and between LR and SCA, but not between IR and SCA. White cell count and hs-CRP were not significantly different between groups, indicating that these individuals did not show evidence of systemic inflammation. Either monocyte subsets shown significant difference between three groups. Next, using flow cytometry assay we analyzed the cell surface LRP1 level in monocyte subsets and total monocytes in individuals of the three groups. Table 2 and Supplementary Figure 2 show that LRP1 was significantly reduced at cell surface in total monocytes of SCA and IR groups respect to LR group; however, non-significant difference was observed between SCA and IR groups. The decreased LRP1 expression was also observed in classical, intermediate and non-classical monocytes in SCA and IR groups respect to LR group.

**TABLE 1:** Clinical and biochemical parameters (Study I).

Parameters	Without subclinical atherosclerosis		Subclinical atherosclerosis (SCA) (*n* = 82)	*P*-value* Binary comparison**
	Low risk group (LR) (*n* = 21)	Intermediate risk group (IR) (*n* = 124)		
Male (%)	6 (28.6)	78 (62.9)	53 (64.6)	0.0073^##^
**Ages, years^#^** Median [IQR]	40 [27–44]	38 [29–47]	50 [44–55]	< 0.0001 ***b, c***
BMI, kg/m^2^ **Mean ± SD**	21.9 ± 2.1	26.5 ± 4.7	26.9 ± 3.7	< 0.0001 ***a, b***
SBP, mmHg Mean ± SD	110 ± 10	118 ± 11	120 ± 12	0.0029 ***a, b***
DBP, mmHg **Mean ± SD**	75 ± 6	82 ± 9	83 ± 7	0.0003 ***a, b***
Total cholesterol, mg/dl Mean ± SD	162 ± 23	191 ± 36	204 ± 34	< 0.0001 ***a, b, c***
LDLc, mg/dl Mean ± SD	89 ± 17	119 ± 31	133 ± 30	< 0.0001 ***a, b, c***
HDLc, mg/dl Mean ± SD	68 ± 18	54 ± 19	49 ± 15	< 0.0001 ***a, b***
Triglycerides, mg/dl Mean ± SD	110 ± 10	116 ± 118	133 ± 76	< 0.0001 ***b, c***
Non-HDLc, mg/dl Mean ± SD	94 ± 18	137 ± 38	155 ± 36	< 0.0001 ***a, b, c***
Lp(a), mg/dl Mean ± SD	41 ± 32	56 ± 60	56 ± 58	*ns*
Glucose, mg/dl Mean ± SD	91 ± 6	98 ± 7	101 ± 9	< 0.0001 ***a, b***
Creatinine, mg/dl Mean ± SD	0.8 ± 0.1	1.0 ± 0.2	1.0 ± 0.2	*ns*
hs-CRP, mg/dl^#^ Median [IQR]	0.11 [0.07–0.14]	0.12 [0.06–0.20]	0.12 [0.10–0.17]	*ns*
White cell count × 10^9^/L^#^ Median [IQR]	6.0 [5.2–6.6]	6.5 [5.5–7.5]	6.4 [5.5–7.8]	*ns*
Classical monocytes^#^, % Median [IQR]	87 [82–89]	86 [83–90]	84 [82–89]	*ns*
Non-classical monocytes^#^,% Median [IQR]	7.0 [6.5–8.0]	7.0 [5.0–9.8]	8.0 [6.0–10.0]	*ns*
Intermediate Monocytes^#^,% Median [IQR]	6.0 [4.0–10.0]	6.0 [5.0–8.0]	7.0 [5.0–8.0]	*ns*
CACS, Agatston units	0	0	> 1.0 F: 5/29 M: 35/53	

*Values are mean ± SD (standard deviation) for normal distribution, or median (IQR, interquartile range) for non-normal distribution*. **BMI**, body mass index; **SBP**, systolic blood pressure; **DBP**, diastolic blood pressure; **LDLc**, low density lipoprotein-cholesterol; **HDLc**, high density lipoprotein-cholesterol; non-HDLc, non-high density lipoprotein-cholesterol; **Lp(a)**, lipoprotein (a); **hs-CRP**, high sensitivity—C-reactive protein; **CACS**, coronary artery calcium score.

^#^Parameters were log-transformed to achieve normal distribution and to apply statistical parametric analysis, but in these table the original data were used.

^##^Contingency analysis and Chi-square test.

*Ordinary one-way ANOVA; ns, non-significant.

**a, **b**, and **c**: umpired t-test for mean values; **a**, IR vs. LR; **b**, SCA vs. LR; and **c**, SCA vs. IR (Letters with significant value, p < 0.05, are shown); **ns**, non-significant p-value.

**TABLE 2:** Comparison of LRP1 expression at cell surface in total monocytes and monocyte subsets from individuals of LR, IR and SCA groups (Study I).

Monocyte LRP1 MFI, arbitrary units	Without subclinical atherosclerosis		Subclinical atherosclerosis (SCA) (*n* = 82)	*P*-value* Binary comparison**
	Low risk group (LR) (*n* = 21)	Intermediate risk group (IR) (*n* = 124)		
Total monocytes Median [IQR]	62.5 [56.9–74.0]	51.9 [43.2–59.1]	48.5 [42.2–58.8]	0.0004 ***a***, *p* = 0.0002 ***b***, *p* < 0.0001 ***c***, *ns*
Classical Monocytes Median [IQR]	67.1 [57.9–78.6]	53.6 [47.4–62.0]	52.4 [44.3–59.7]	0.0002 ***a***, *p* = 0.0001 ***b***, *p* < 0.0001 ***c***, *ns*
Intermediate Monocytes Median [IQR]	88.2 [70.9–103.0]	69.0 [60.0–84.6]	68.1 [60.9–82.3]	0.0083 ***a***, *p* = 0.0024 ***b***, *p* < 0.0001 ***c***, *ns*
Non-classical Monocytes Median [IQR]	49.2 [33.1–58.9]	36.6 [30.4–45.3]	35.0 [30.0–44.8]	0.0206 ***a***, *p* = 0.0088 ***b***, *p* = 0.0073 ***c***, *ns*

Values are median (IQR, interquartile range). Parameters were log-transformed to achieve normal distribution and to apply statistical parametric analysis, but in these table the original data were used.

*Ordinary one-way ANOVA; ns, non-significant.

**a, **b**, and **c**: umpired t-test for mean values; **a**, IR vs. LR; **b**, SCA vs. LR; and **c**, SCA vs. IR. Significant value, p < 0.05. **MFI**, mean fluorescence intensity.

Considering that ages and plasma triglycerides were not significantly different between LR and IR groups (Table 1) can be concluded that these parameters are not related with the decreased LRP1 expression at the cell surface of monocytes observed in individuals with IR and SCA. Moreover, the LRP1 expression in monocytes remained statistically decreased in IR and SCA groups with respect to the LR group when these values were statistically adjusted for BMI, SBP, DBP, glucose, and lipid parameters (data not shown). In this way, the expression level of LRP1 at the cell surface of total monocytes only showed significant correlation for total cholesterol and non-HDLc but not for BMI, triglycerides and LDLc when all individuals were included in the statistical analysis (*n* = 227). None of these parameters showed significant correlations with LRP1 levels when in each group was evaluated (Supplementary Table 3).

Additional monocytes cell surface markers were also analyzed in comparison with cell surface LRP1 in the three groups. The expression of CD36, a scavenger receptor of oxidized LDL (19), and CD11b and CD11c, integrin complexes involved in cell adhesion of monocytes (20), were measured using flow cytometry assay following the strategy showed in Supplementary Figure 1. Both CD36 and CD11b were mainly expressed in total, intermediate and classical monocytes, whereas CD11c was highly expressed in non-classical and intermediate monocytes in individuals of LR group (Supplementary Figure 3). However, none of these cell surface marker proteins showed significant differences between three groups in total monocytes nor monocyte subsets (Supplementary Table 4).

**FIGURE 1:**
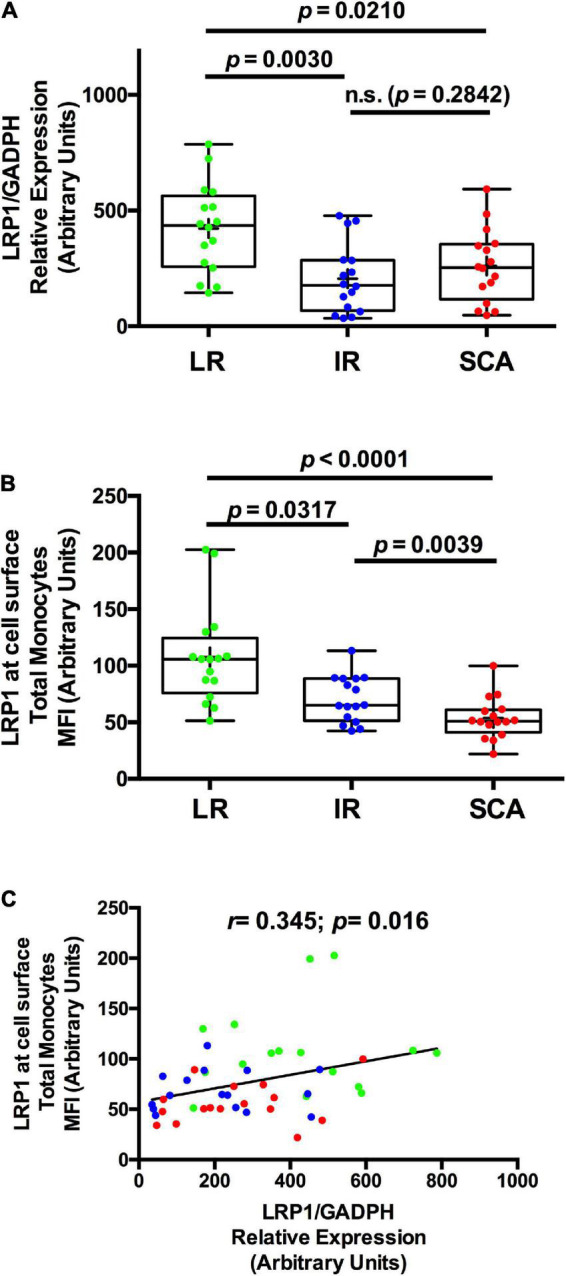
*LRP1* gene expression is also decreased in individuals with SCA and IR. **(A)** The levels of LRP1 specific transcripts were measured by quantitative RT-PCR (qPCR) in FACS-isolated total monocytes as was described in “Materials and Methods” section and relatively expressed at *GAPDH* levels from individuals with low risk (LR) (green symbols; *n* = 16), intermediate risk (IR) (blue symbols; *n* = 16) and subclinical atherosclerosis (SCA) (red symbols; *n* = 16) (Study II). **(B)** The LRP1 expression level at cell surface was measured by flow cytometry assays in total monocytes from peripheral blood extracted of individuals with low risk (LR) (green symbols; *n* = 16), intermediate risk (IR) of CVD (blue symbols; *n* = 16), and subclinical atherosclerosis (SCA) (red symbols; *n* = 16). In **(A,B)**, results are shown as box plot representing the median value and interquartile range (IQR) of arbitrary units for qPCR and MIF (mean intensity of fluorescence), respectively. Maximal and minimal values for each study group are indicated (bar lines). Parameters were log-transformed to achieve normal distribution and to apply statistical parametric analysis, but in these table the original data were used. **(C)** Correlation analysis between LRP1 protein and *LRP1* mRNA in monocytes of individuals with LR (green symbols), IR (blue symbols) and SCA (red symbols). *p*-values are shown and differences were considered statistically significant when *p* < 0.05.

To evaluate whether the reduced expression of LRP1 at cell surface in total monocyte correlates with the *LRP1* gene expression, we analyzed *LRP1* mRNA levels in total monocytes isolated by FACS from peripheral blood of sixteen SCA, IR, and LR individuals (Study II). The clinical and biochemistry parameters of the three groups (Study II) are summarized in Supplementary Table 5, which showed similar results to those parameters showed in Study I. Figure 1A shows that *LRP1* mRNA level in FACS-isolated total monocytes decreased in SCA and IR groups respect to LR group. In agreement with results of the Study I, LRP1 expression at cell surface in total monocytes, measured by flow cytometry, was significantly reduced in SCA and IR groups compared to LR group (Figure 1B). The correlation analysis between *LRP1* mRNA and LRP1 protein expressed at cell surface in total monocytes for three groups were significant (*p* = 0.016) but the value of *r* was substantially less than 1 (*r* = 0.345) (Figure 1C), suggesting that changes of *LRP1* transcripts are not strongly associated with LRP1 protein levels in the three groups studied.

**FIGURE 2:**
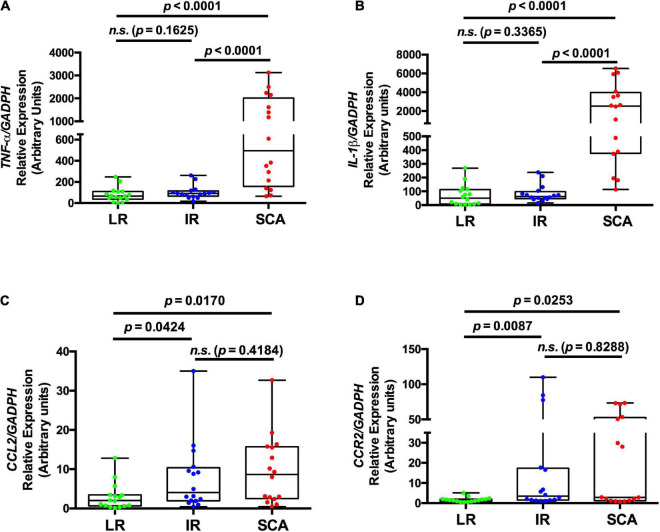
Gene expression of pro-inflammatory factors is increased in circulating monocytes in SCA. The levels of specific transcripts of *TNF-*α **(A)**, *IL-1*β **(B)**, *CCL2* **(C)**, and *CCR2* **(D)** were measured by quantitative RT-PCR (qPCR) in FACS-isolated total monocytes and relatively expressed at *GAPDH* levels from individuals with low risk (LR) (green symbols; *n* = 16), intermediate risk (IR) (blue symbols; *n* = 16) and subclinical atherosclerosis (SCA) (red symbols; *n* = 16) (Study II). Results are shown as box plot representing the median value and interquartile range (IQR) of arbitrary units for qPCR. Maximal and minimal values for each study group are indicated (bar lines). Parameters were log-transformed to achieve normal distribution and to apply statistical parametric analysis, but in these table the original data were used. *p*-values are shown and differences were considered statistically significant when *p* < 0.05.

Previous reports showed that the LRP1 deficiency in mouse myeloid cells induced the release of pro-inflammatory factors, such as TNF-α, IL-1β, and CCL2 (16). Moreover, it has been shown that circulating monocytes acquire pro-inflammatory profiles in early steps of the atherosclerosis development (6, 21). Thus, we determined the expression of pro-inflammatory factors in FACS-isolated total monocytes of SCA, IR, and LR groups to evaluate the associations with LRP1 expression in these cells (Study II). Figures 2A–D shows increased mRNA levels of *TNF-*α and *IL-1*β (*p* < 0.0001) in total monocytes of SCA group respect to IR and LR groups. No differences were observed between LR and IR groups. In addition, *CCL2* and *CCR2* expressions were significantly increased in SCA and IR groups with respect to LR group, whereas non-differences were observed between SCA and IR groups. Next, we evaluated whether the cell surface LRP1 expression in total monocytes is associated with expression levels of pro-inflammatory factors in total monocytes in the three groups (Study II). In Supplementary Table 6 is observed that cell surface LRP1 in total monocytes showed significant negative correlation with mRNA levels of *TNF-*α and *IL-1*β, but not with *CCL2* and *CCR2* when all individuals were included in this analysis (*n* = 48). On the other hand, no statistical significance in the correlation analysis were found between *LRP1* mRNA and these pro-inflammatory factors in circulating total monocytes. Finally, in Supplementary Figure 4 is deduced that 10/16 individuals of SCA group presented high levels of *TNF-*α and *IL-1*β, which were associated with low LRP1 expression in total monocytes in 13/16 individuals (low limit of IQR with MFI values < 76 as is indicated in Figure 1B). On the other hand, whole LR and IR group showed low levels of these pro-inflammatory factors, whereas low LRP1 expression was observed in 1/16 individuals with LR and 7/16 individuals with IR.

## Discussion

Several studies have shown that LRP1 plays a dual role in the development and regression of atherosclerosis (9, 22). In VSMCs, LRP1 mediates the foam cells formation through the uptake of aggregated LDL (aggLDL) inducing intracellular cholesteryl ester accumulation (23, 24). On the other hand, LRP1 can help to suppress atherosclerosis by inhibiting the PDGF-β signaling pathway and blocking the cell proliferation of VSMCs (25). A similar dual role it has been shown in macrophages in which LRP1 could mainly have an atheroprotective effect mainly by decreasing inflammation, facilitating efferocytosis and promoting cholesterol efflux (9, 22), but it also promotes the macrophages-derived foam cell formation through the binding and internalization of aggLDL (26, 27). In the present study, we found decreased LRP1 levels at cell surface in total monocytes and monocyte subsets, particularly in classical monocytes, in asymptomatic individuals with CVD risk factors (IR group) and with atherosclerotic plaque detected by carotid ultrasound (CU) and CAC score > 1 (SCA group) respect to individuals with low CVD risk factors (LR group).

It is well established that classical monocytes are involved in the early stages of atherosclerosis by its ability to interact with activated endothelial cell adhesion molecules through membrane integrin complexes expressed at cell surface of this monocyte subset (28). Several monocytic integrins, included CD11b and CD11c, facilitate the adhesion, rolling and extravasation processes of monocytes to vessel intima and macrophage differentiation (20, 29). In macrophages, LRP1 located at plasma membrane plays a key role in the regulation of integrins promoting the migratory processes (30). Moreover, circulating monocytes express increased CD36 levels in individuals with high risk to develop atherosclerosis (31). In the present study we found that CD11c was highly expressed in non-classical and intermediate monocytes in LR group, whereas CD11b and CD36 were mainly expressed in classical monocytes, which agree with results previously published (32). However, the cell surface levels of these monocytic markers showed no significant changes between three groups studied. The absence of modifications in CD11c, CD11b, and CD36 levels in IR and SCA groups suggest a very early stage in the development of atherosclerosis in these asymptomatic individuals. In this way, we found that decreased LRP1 expression at cell surface in total monocytes only correlated with total cholesterol and non-HDLc but not with LDLc in the totality of individuals studied, which may be related with the moderate increase of plasma LDLc observed in these subjects. In addition, although the age is a known risk factor for atherosclerosis development (1, 2), in our study we observed that the age would be an independent factor for the LRP1 expression in total monocytes since it is decreased in IR group but not in LR group with comparable ages in both groups. Taking into account our data, reduced levels of LRP1 at cell surface in circulating monocytes could be considered as a risk factor of atherosclerosis development in asymptomatic individuals.

Together with decreased LRP1 expression at cell surface also we found a significant decrease in *LRP1* mRNA expression in total monocytes of SCA and IR groups with respect to LR group. However, the LRP1 protein levels showed a poor correlation with LRP1 mRNA expression in the totality of individuals, which could be related to different analytical variations between assays together other aspects referred with the regulation of gene expression and protein stability of LRP1. Different studies have reported that the regulation of *LRP1* gene expression is very complex and dependent on the cell type (26, 33, 34). In addition, LRP1 has a relative long protein stability respect to other members of LDL receptor family (35). In this study, decreased levels of LRP1 expression at cell surface in monocytes of SCA and IR groups may be related to an accelerated degradation of LRP1 (35). In this way, different extracellular factors, such as LPS, insulin and hemin, promote the LRP1 degradation through the activation of proteasomal and lysosomal pathways in macrophages and other cells (13, 36, 37). Finally, decreased LRP1 expression at cell surface in monocytes may be due to an increased shedding events in LRP1 at plasma membrane, like those that occur in inflammation by the action of LPS and IFN-γ in macrophages (9). The loss of LRP1 at cell surface has functional consequences since this receptor regulates other membrane proteins, such as PDGF-R (38), insulin receptor (39), urokinase receptor (40) and Toll-like (TL) receptors (15), which are tightly involved with the vascular integrity, glucose homeostasis, cell migration, and inflammation, respectively. Taking into account that atherosclerosis is an inflammatory process, additional studies are needed to determine the association between LRP1 expression at cell surface in total monocytes with the atherosclerosis development.

It has been shown that LRP1 is involved in inflammatory processes based on its ability to regulate the release of pro-inflammatory factors, such as TNF-α, IL-6, and CCL2, in macrophages (16). Thus, LRP1 modulates the activation of TL-receptors, mainly TLR2 and TLR4, through a subsequent recruit of Rab8a/PI3K complex, which promotes the suppression of pro-inflammatory factor release (15). In this way, the downregulation of LRP1 protein by specific *LRP1* gene deletion produce a pro-inflammatory profile in macrophages (16). Our results showed that decreased LRP1 expression at cell surface in total monocytes, but not *LRP1* mRNA, is associated with a monocyte pro-inflammatory profile in individuals with SCA, characterized by increased levels of *TNF-*α and *IL-1*β mRNA, which could contribute to the persistent activation of monocytes as mediators of atherosclerosis development (7).

Some limitations are noteworthy. First, our results support the fact that the LRP1 expression at cell surface of monocytes may be a better indicator of SCA. However, the low number of individuals with low risk factor of CVD studied is insufficient to corroborate this role as an epidemiological biomarker and more extensive intervention studies are needed. Second, the measurement of pro-inflammatory factors and other markers in total monocytes of peripheral blood is very complex and may produce significant variations. Thus, the validation of these results in isolated mononuclear cells of peripheral blood by Ficoll-Hypaque solution could facilitate these measurements reducing the analytical variation of total results. Finally, other subclinical conditions or diseases that were not recorded in the present study may have effect on the expression level of LRP1 in total monocytes with pro-inflammatory profile.

In summary, we propose that the decreased LRP1 expression at cell surface in total monocytes with pro-inflammatory profile is associated with the development of atherosclerosis in asymptomatic individuals. Our results taken together have important diagnostic and therapeutic connotations related to SCA. Future large prospective cohort and intervention studies are required to confirm these findings, in particular to study LRP1 expression in circulating monocytes in individuals that have risk factors of CVD but are not classified as SCA according to image criteria for atherosclerosis used in the present work.

## Data availability statement

The original contributions presented in this study are included in the article/Supplementary material, further inquiries can be directed to the corresponding author/s.

## Ethics statement

The studies involving human participants were reviewed and approved by the CIEIS HP 4-178 Hospital Privado Universitario de Cordoba. The patients/participants provided their written informed consent to participate in this study.

## Author contributions

RA, JN, RC, and GC designed the research. DF contributed with the flow cytometry assays. MT performed and analyzed the image studies. RC and GC contributed new reagents and analytic tools. VA and GC analyzed the data and wrote the manuscript. All authors contributed to the article and approved the submitted version.

## Conflict of interest

The authors declare that the research was conducted in the absence of any commercial or financial relationships that could be construed as a potential conflict of interest.

## Publisher’s note

All claims expressed in this article are solely those of the authors and do not necessarily represent those of their affiliated organizations, or those of the publisher, the editors and the reviewers. Any product that may be evaluated in this article, or claim that may be made by its manufacturer, is not guaranteed or endorsed by the publisher.

## References

[1] Peters SAE den Ruijter HM Bots ML Moons KGM. Improvements in risk stratification for the occurrence of cardiovascular disease by imaging subclinical atherosclerosis: a systematic review. *Heart.* (2012) 98:177–84. 10.1136/heartjnl-2011-300747 22095617

[2] Albertini RA Ferrer DG Romagnoli PA Tinti ME Amigone JL Capra R Association between cardiovascular disease risk scores and subclinical atherosclerosis prevalence in non-elderly adult patients from Argentina. *Int J Cardiovasc Imaging.* (2017) 33:1521–9. 10.1007/s10554-017-1152-9 28493105

[3] Polak JF Pencina MJ Herrington D O’Leary DH. Associations of edge-detected and manual-traced common carotid intima-media thickness measurements with framingham risk factors: the multi-ethnic study of atherosclerosis. *Stroke.* (2011) 42:1912–6. 21546477 10.1161/STROKEAHA.110.603449 PMC3169166

[4] Wolf D Ley K. Immunity and inflammation in atherosclerosis. *Circ Res.* (2019) 124:315–27. 30653442 10.1161/CIRCRESAHA.118.313591 PMC6342482

[5] Bernal-Lopez MR Llorente-Cortes V Calleja F Lopez-Carmona D Mayas MD Gomez-Huelgas R Effect of different degrees of impaired glucose metabolism on the expression of inflammatory markers in monocytes of patients with atherosclerosis. *Acta Diabetol.* (2013) 50:553–62. 10.1007/s00592-011-0337-2 21997325

[6] Bekkering S van den Munckhof I Nielen T Lamfers E Dinarello C Rutten J Innate immune cell activation and epigenetic remodeling in symptomatic and asymptomatic atherosclerosis in humans in vivo. *Atherosclerosis.* (2016) 254:228–36. 10.1016/j.atherosclerosis.2016.10.019 27764724

[7] Zhong C Yang X Feng Y Yu J. Trained immunity: an underlying driver of inflammatory atherosclerosis. *Front Immunol.* (2020) 21:284. 10.3389/fimmu.2020.00284 32153588 PMC7046758

[8] Herz J Strickland DK. LRP: a multifunctional scavenger and signaling receptor. *J Clin Invest.* (2001) 108:779–84. 11560943 10.1172/JCI13992 PMC200939

[9] Actis Dato V Chiabrando G. The role of low-density lipoprotein receptor-related protein 1 in lipid metabolism, glucose homeostasis and inflammation. *Int J Mol Sci.* (2018) 19:1780. 10.3390/ijms19061780 PMC6032055 29914093

[10] Boucher P Herz J. Signaling through LRP1: protection from atherosclerosis and beyond. *Biochem Pharmacol.* (2011) 81:1–5. 10.1016/j.bcp.2010.09.018 20920479 PMC2991482

[11] Xian X Ding Y Dieckmann M Zhou L Plattner F Liu M LRP1 integrates murine macrophage cholesterol homeostasis and inflammatory responses in atherosclerosis. *Elife.* (2017) 6:e29292. 10.7554/eLife.29292 29144234 PMC5690284

[12] Cáceres LC Bonacci GR Sánchez MC Chiabrando GA. Activated α2 macroglobulin induces matrix metalloproteinase 9 expression by low-density lipoprotein receptor-related protein 1 through MAPK-ERK1/2 and NF-κB activation in macrophage-derived cell lines. *J Cell Biochem.* (2010) 111:607–17. 10.1002/jcb.22737 20568116

[13] Bonacci GR Cáceres LC Sánchez MC Chiabrando GA. Activated α2-macroglobulin induces cell proliferation and mitogen-activated protein kinase activation by LRP-1 in the J774 macrophage-derived cell line. *Arch Biochem Biophys.* (2007) 460:100–6. 10.1016/j.abb.2007.01.004 17288987

[14] Bornachea O Benitez-Amaro A Vea A Nasarre L de Gonzalo-Calvo D Escola-Gil JC Immunization with the Gly1127-Cys1140 amino acid sequence of the LRP1 receptor reduces atherosclerosis in rabbits. Molecular, immunohistochemical and nuclear imaging studies. *Theranostics.* (2020) 10:3263–80. 10.7150/thno.37305 32194867 PMC7053206

[15] Luo L Wall AA Tong SJ Hung Y Xiao Z Tarique AA TLR crosstalk activates LRP1 to recruit rab8a and PI3Kγ for suppression of inflammatory responses. *Cell Rep.* (2018) 24:3033–44. 10.1016/j.celrep.2018.08.028 30208326

[16] Mantuano E Brifault C Lam MS Azmoon P Gilder AS Gonias SL. LDL receptor-related protein-1 regulates NFκB and microRNA-155 in macrophages to control the inflammatory response. *Proc Natl Acad Sci U.S.A.* (2016) 113:1369–74. 26787872 10.1073/pnas.1515480113 PMC4747752

[17] Ferrer DG Jaldín-Fincati JR Amigone JL Capra RH Collino CJ Albertini RA Standardized flow cytometry assay for identification of human monocytic heterogeneity and LRP1 expression in monocyte subpopulations: decreased expression of this receptor in nonclassical monocytes. *Cytometry A.* (2014) 85:601–10. 10.1002/cyto.a.22455 24639232

[18] Fraser GG Harris EK. Generation and application of data on biological variation in clinical chemistry. *Crit Rev Clin Lab Sci.* (1989) 27:409–37. 2679660 10.3109/10408368909106595

[19] Kunjathoor VV Febbraio M Podrez EA Moore KJ Andersson L Koehn S Scavenger receptors class A-I/II and CD36 are the principal receptors responsible for the uptake of modified low density lipoprotein leading to lipid loading in macrophages. *J Biol Chem.* (2002) 277:49982–8. 12376530 10.1074/jbc.M209649200

[20] Schittenhelm L Hilkens CM Morrison VL. β2 integrins as regulators of dendritic cell, monocyte, and macrophage function. *Front Immunol.* (2017) 8:1866. 10.3389/fimmu.2017.01866 29326724 PMC5742326

[21] Christ A Bekkering S Latz E Riksen NP. Long-term activation of the innate immune system in atherosclerosis. *Semin Immunol.* (2016) 28:384–93. 27113267 10.1016/j.smim.2016.04.004

[22] Chen J Su Y Pi S Hu B Mao L. The dual role of low-density lipoprotein receptor-related protein 1 in atherosclerosis. *Front Cardiovasc Med.* (2021) 8:682389. 10.3389/fcvm.2021.682389 34124208 PMC8192809

[23] Costales P Fuentes-Prior P Castellano J Revuelta-Lopez E Corral-Rodríguez MÁ Nasarre L K domain CR9 of low density lipoprotein (LDL) receptor-related protein 1 (LRP1) is critical for aggregated LDL-induced foam cell formation from human vascular smooth muscle cells. *J Biol Chem.* (2015) 290:14852–65. 10.1074/jbc.M115.638361 25918169 PMC4463433

[24] Camino-López S Llorente-Cortés V Sendra J Badimon L. Tissue factor induction by aggregated LDL depends on LDL receptor-related protein expression (LRP1) and Rho A translocation in human vascular smooth muscle cells. *Cardiovasc Res.* (2007) 73:208–16. 10.1016/j.cardiores.2006.10.017 17141748

[25] Zhou L Takayama Y Boucher P Tallquist MD Herz J. LRP1 regulates architecture of the vascular wall by controlling PDGFRβ-dependent phosphatidylinositol 3-kinase activation. *PLoS One.* (2009) 4:e6922. 10.1371/journal.pone.0006922 19742316 PMC2734324

[26] Llorente-Cortés V Royo T Otero-Viñas M Berrozpe M Badimon L. Sterol regulatory element binding proteins downregulate LDL receptor-related protein (LRP1) expression and LRP1-mediated aggregated LDL uptake by human macrophages. *Cardiovasc Res.* (2007) 74:526–36. 10.1016/j.cardiores.2007.02.020 17376415

[27] Llorente-Cortés V Royo T Juan-Babot O Badimon L. Adipocyte differentiation-related protein is induced by LRP1-mediated aggregated LDL internalization in human vascular smooth muscle cells and macrophages. *J Lipid Res.* (2007) 48:2133–40. 10.1194/jlr.M700039-JLR200 17620659

[28] Zawada AM Rogacev KS Rotter B Winter P Marell RR Fliser D SuperSAGE evidence for CD14 ++CD16 + monocytes as a third monocyte subset. *Blood.* (2011) 118:e50–61. 10.1182/blood-2011-01-326827 21803849

[29] Barry OP Praticò D Savani RC FitzGerald GA. Modulation of monocyte-endothelial cell interactions by platelet microparticles. *J Clin Invest.* (1998) 102:136–44. 10.1172/JCI2592 9649567 PMC509075

[30] Ferrer DG Dato VA Fincati JRJ Lorenc VE Sánchez MC Chiabrando GA. Activated α-macroglobulin induces mesenchymal cellular migration of Raw264.7 cells through low-density lipoprotein receptor-related protein 1. *J Cell Biochem.* (2017) 118:1810–8. 10.1002/jcb.25857 28012205

[31] Bernal-Lopez RM Llorente-Cortes V López-Carmona D Mayas DM Gomez-Huelgas R Tinahones FJ Modulation of human monocyte CD36 by type 2 diabetes mellitus and other atherosclerotic risk factors. *Eur J Clin Invest.* (2011) 41:854–62. 10.1111/j.1365-2362.2011.02475.x 21668445

[32] Thomas GD Hamers AAJ Nakao C Marcovecchio P Taylor AM McSkimming C Human blood monocyte subsets. *Arterioscler Thromb Vasc Biol.* (2017) 37:1548–58. 28596372 10.1161/ATVBAHA.117.309145 PMC5828170

[33] Costales P Aledo R Vérnia S Das A Shah VH Casado M Selective role of sterol regulatory element binding protein isoforms in aggregated LDL-induced vascular low density lipoprotein receptor-related protein-1 expression. *Atherosclerosis.* (2010) 213:458–68. 10.1016/j.atherosclerosis.2010.09.034 20980003

[34] Costales P Castellano J Revuelta-López E Cal R Aledo R Llampayas O Lipopolysaccharide downregulates CD91/low-density lipoprotein receptor-related protein 1 expression through SREBP-1 overexpression in human macrophages. *Atherosclerosis.* (2013) 227:79–88. 23312784 10.1016/j.atherosclerosis.2012.12.021

[35] Melman L Geuze HJ Li Y McCormick LM van Kerkhof P Strous GJ Proteasome regulates the delivery of LDL receptor-related protein into the degradation pathway. *Mol Biol Cell.* (2002) 13:3325–35. 10.1091/mbc.e02-03-0152 12221136 PMC124162

[36] Grosso RA Caldarone PVS Sánchez MC Chiabrando GA Colombo MI Fader CM. Hemin induces autophagy in a leukemic erythroblast cell line through the LRP1 receptor. *Biosci Rep.* (2019) 39:BSR20181156. 10.1042/BSR20181156 30523204 PMC6328880

[37] Ceschin DG Sánchez MC Chiabrando GA. Insulin induces the low density lipoprotein receptor-related protein 1 (LRP1) degradation by the proteasomal system in J774 macrophage-derived cells. *J Cell Biochem.* (2009) 106:372–80. 10.1002/jcb.22014 19115269

[38] Strickland DK Au DT Cunfer P Muratoglu SC. Low-density lipoprotein receptor-related protein-1: role in the regulation of vascular integrity. *Arterioscler Thromb Vasc Biol.* (2014) 34:487–98. 24504736 10.1161/ATVBAHA.113.301924 PMC4304649

[39] Actis Dato V Benitez-Amaro A de Gonzalo-Calvo D Vazquez M Bonacci G Llorente-Cortés V LRP1-mediated AggLDL endocytosis promotes cholesteryl ester accumulation and impairs insulin response in HL-1 cells. *Cells.* (2020) 9:182. 10.3390/cells9010182 31936892 PMC7016900

[40] Gonias SL. Plasminogen activator receptor assemblies in cell signaling, innate immunity, and inflammation. *Am J Physiol Cell Physiol.* (2021) 321:C721–34. 10.1152/ajpcell.00269.2021 34406905 PMC8560384

