# Supplementary material for: Decreased low-density lipoprotein receptor-related protein 1 expression in pro-inflammatory monocytes is associated with subclinical atherosclerosis
Source: Front Cardiovasc Med. 2022 Jul 26;9:949778. doi: 10.3389/fcvm.2022.949778 (PMC9360420; doi:10.3389/fcvm.2022.949778)
Supplement: Supplementary file 1 [file Data_Sheet_1.PDF]

Supplementary Methods.

2.1. Study participants and study design

Inclusion criteria:

Low risk (LR) criteria: plasma total cholesterol < 200 mg/dl, glucose < 100 mg/dl, HDLc ≥ 40 mg/dl in males and ≥ 50 mg/dl in females, triglycerides < 150 mg/dl, creatinine < 1,2 mg/dl, systolic blood pressure (SBP) < 140 mm Hg, diastolic blood pressure (DBP) < 90 mm Hg, body mass index(BMI) <25 kg/m2 and normal carotid ultrasound and coronary artery calcium score, CAC =0.

SCA criteria: asymptomatic individuals with carotid atherosclerotic plaque and/or coronary artery calcium score, CAC >0, independent of the presence or not of other risk factors of CVD found.

Intermediate risk (IR) criteria: asymptomatic individuals with at least one of the factors included in LR criteria and without carotid atherosclerotic plaque and/or coronary artery calcium score, CAC >0.

Exclusion criteria:

Individuals with arterial hypertension antecedents, with current smoking or at least 5 years of current smoking abstinence, history of early familial coronary artery disease, known sleep apnea and hypothyroidism, use of oral contraceptives, hormone replacement therapy or finasteride in the past month.

2.2. Carotid ultrasound (CU) study

The distal segment of the common carotid artery, the carotid bulb and the proximal segment of the internal carotid artery were examined. The results from the CU were considered normal when CIMT was <0.9 mm and abnormal when CIMT was >0.9 mm and <1.5 mm. However, the presence of carotid atherosclerotic plaque was defined by: (a) abnormal structure (lumen protrusions and loss of alignment between adjacent walls), and (b) abnormal wall echogenicity following the criteria of the Atherosclerosis Risk in Communities (ARIC) [1,2]. The same imaging expert of the HPUC performed all CU procedures under blinded conditions without identifying to which risk group the individual belonged.

The distal segment of the common carotid artery, the carotid bulb and the proximal segment of the internal carotid artery were examined. The results from the CU were considered abnormal when an increased CIMT (>0.9 mm) or a carotid atherosclerotic

plaque was detected. However, the presence of carotid atherosclerotic plaque was defined by: (a) abnormal structure (lumen protrusions and loss of alignment between adjacent walls), and (b) abnormal wall echogenicity following the criteria of the Atherosclerosis Risk in Communities (ARIC) study [1]. The same imaging expert of the HPUC performed all CU procedures.

2.3. Coronary artery calcium (CAC) score determination by cardiac computed tomography (CT)

The human chorionic gonadotropin (HCG) was assayed to detect pregnancy in females before performing Cardiac CT and only HCG-negative individuals were included in the study. All Cardiac CT procedures were performed by the same imaging expert of the HPUC using software validated to score the CAC. Based on the number of calcified lesions found in each subject, the CAC was determined using the Agatston method [3]. An abnormal CAC score was defined as >0.

2.5. Flow cytometry assays

CD45-positive leucocytes were visualized in SSC versus CD45 plots showing all fluorescence-3 (PC5-positive) events using a BD FACSCalibur™ Flow Cytometer for Study I and BD FACSCanto™II flow cytometer (BD Biosciences, San Jose, CA) for Study II in which both equipment were calibrated with BD Cytometer Setup Tracking Beads (No. 641319, BD Biosciences) and Accudrop Beads (No. 345248, BD Biosciences). BD FACSDivaTM software (version 6.1.2) was used to acquire and analyse the data as was previously described [4]. In *Supplementary Figure S1* is represented the flow cytometry strategy used. Briefly, an acquisition threshold, which was set such that any unwanted events like CD45-negative platelets, dead cells, and debris were not recorded. The monocytes were then defined by sequential gating on all CD45-positive leukocytes using the SSC versus LRP1-staining plot (PE), whereas monocyte subpopulations were identified from CD14 (FITC) versus CD16 (APC-Cy7) plot following the criteria previously defined [4,5]. Using isotype controls, voltage, and compensation, the instrument was set such that the cells were adequately positioned in the dot plots. The mean fluorescence intensity (MFI) for LRP1, CD36, CD11b, and CD11c in classical, intermediate, and non-classical monocytes was determined from the cell distribution pattern obtained in the CD14 versus CD16 plot, whereas in total

monocytes was defined from cells gated on SSC versus LRP1-positive plot (*Supplementary Figure S1*) [4].

2.7. Quantitative reverse transcriptase-PCR assays

To evaluate the specific mRNA for each parameter, total monocytes isolated were treated with TRIzolVR Reagent (Invitrogen, Buenos Aires, Argentina). Total RNAs were extracted from samples by a single step method using a RNeasy Mini-Kit (Qiagen, Chatsworth, CA), according to the manufacturer's instructions. A reverse transcription polymerase chain reaction (RT-PCR) was used to measure the transcript level of LRP1 and pro-inflammatory factors as was previously described [6]. Briefly, templates of total RNA were obtained using random hexaprimers. Templates of total RNA were obtained using random hexaprimers. Specific primers for *LRP1*, *TNF-α*, *IL-1β*, *CCL2*, *CCR2* and *GAPDH* are indicated in Supplementary Table S2. PCR conditions were optimized to evaluate all transcripts together. *GAPDH* was used as a housekeeping gene and the results were normalized to RT-PCR products of *GAPDH* transcripts. Transcripts were quantified by real-time qRT-PCR (ABI 7500 Sequence Detection System, Applied Biosystems, Foster City, CA) using Sequence Detection software v1.4. Cycling were a warm start at 95 °C for 10 min, followed by 40 cycles at 95 °C for 15 s and 60 °C for 1 min. Relative gene expression was calculated by the 2-Ct method. Each sample was analysed in triplicate. No amplification was observed using water or RNA samples incubated without reverse transcriptase during cDNA synthesis.

References

[1] L.E. Chambless, G. Heiss, A.R. Folsom, W. Rosamond, M. Szklo, A.R. Sharrett, L.X. Clegg, Association of coronary heart disease incidence with carotid arterial wall thickness and major risk factors: The Atherosclerosis Risk in Communities (ARIC) study, 1987-1993, American Journal of Epidemiology. 146 (1997). https://doi.org/10.1093/oxfordjournals.aje.a009302.

[2] P. Raggi, J.H. Stein, Carotid intima-media thickness should not be referred to as subclinical atherosclerosis: A recommended update to the editorial policy at Atherosclerosis, Atherosclerosis. 312 (2020). https://doi.org/10.1016/j.atherosclerosis.2020.09.015.

[3] A.S. Agatston, W.R. Janowitz, F.J. Hildner, N.R. Zusmer, M. Viamonte, R. Detrano, Quantification of coronary artery calcium using ultrafast computed tomography, J Am Coll Cardiol. 15 (1990). https://doi.org/10.1016/0735-1097(90)90282-T.

[4] D.G. Ferrer, J.R. Jaldín-Fincati, J.L. Amigone, R.H. Capra, C.J. Collino, R.A. Albertini, G.A. Chiabrando, Standardized flow cytometry assay for identification

of human monocytic heterogeneity and LRP1 expression in monocyte subpopulations: Decreased expression of this receptor in nonclassical monocytes, Cytometry Part A. 85 (2014). https://doi.org/10.1002/cyto.a.22455.

[5] L. Ziegler-Heitbrock, P. Ancuta, S. Crowe, M. Dalod, V. Grau, D.N. Hart, P.J.M. Leenen, Y.J. Liu, G. MacPherson, G.J. Randolph, J. Scherberich, J. Schmitz, K. Shortman, S. Sozzani, H. Strobl, M. Zembala, J.M. Austyn, M.B. Lutz, Nomenclature of monocytes and dendritic cells in blood, Blood. 116 (2010). https://doi.org/10.1182/blood-2010-02-258558.

[6] V. Actis Dato, A. Benitez-Amaro, D. de Gonzalo-Calvo, M. Vazquez, G. Bonacci, V. Llorente-Cortés, G.A. Chiabrando, LRP1-Mediated AggLDL Endocytosis Promotes Cholesteryl Ester Accumulation and Impairs Insulin Response in HL-1 Cells, Cells. 9 (2020) 182. https://doi.org/10.3390/cells9010182.
