# Supplementary material for: Decreased low-density lipoprotein receptor-related protein 1 expression in pro-inflammatory monocytes is associated with subclinical atherosclerosis
Source: Front Cardiovasc Med. 2022 Jul 26;9:949778. doi: 10.3389/fcvm.2022.949778 (PMC9360420; doi:10.3389/fcvm.2022.949778)
Supplement: Supplementary file 2 [file Data_Sheet_2.PDF]

Supplementary Figures

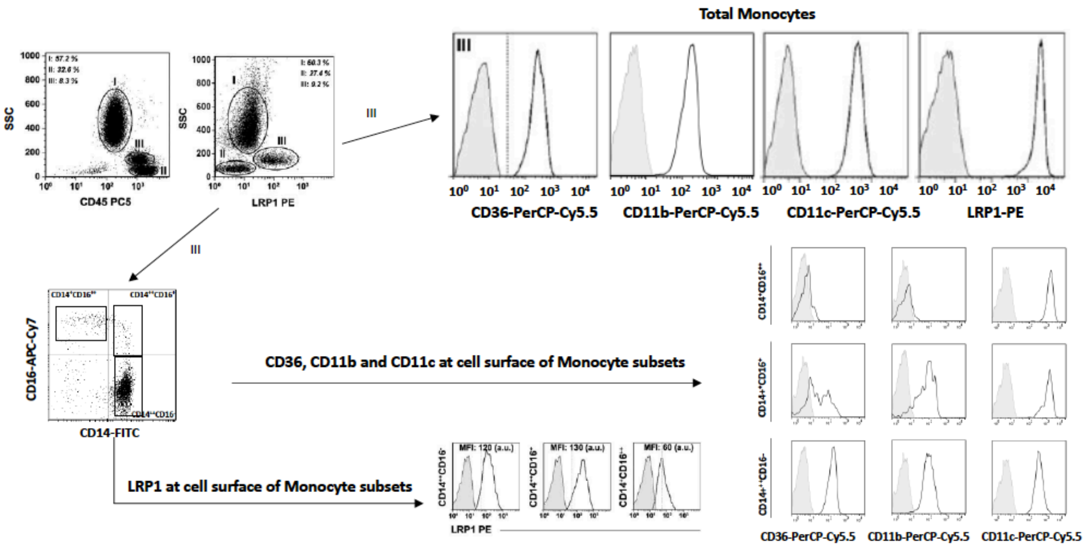

Supplementary Figure S1. ***Flow cytometry using the gating strategy LRP1 to measure LRP1, CD36, CD11b, and CD11c expressions at cell surface in total monocytes and monocyte subsets***. CD45-positive leucocytes were visualized in SSC vs. CD45 plot showing all fluorescence-3 (PC5-positive) events. An acquisition threshold was set such that any unwanted events like CD45-negative platelets, dead cells, and debris were not recorded. The monocytes (III) were then defined by sequential gating on all CD45-positive leukocytes using the SSC vs. LRP1-staining plot, whereas monocyte subpopulations were identified from CD14 vs. CD16 plot. Using isotype controls, voltage and compensation, the instrument was set for the cells to be adequately positioned in the dot plots. The mean fluorescence intensity (MFI) for LRP1, CD36, CD11b, and CD11c in total monocytes (III) was determined in the SSC vs. LRP1 plot, whereas in classical, intermediate, and non-classical monocyte subsets was determined from the cell distribution pattern obtained in the CD14 vs. CD16 plot.

FACS plots for cell sorting of total monocytes. Fresh whole blood was drawn into EDTA-K3 collection tubes (DVS, N° 1213752.5, Buenos Aires, Argentina) and specimens were prepared for flow cytometry within 30 min. Whole blood (6 ml) was treated with lysing buffer (N° 555899; BD Pharm Lyse) for 20 minutes. Washed pellet cells were incubated with each antigen specific fluorochrome-labeled antibody (dilution 1:50). Samples were then incubated for 20 min at 4 °C in the dark. To ensure maximum viability, stained cells were cell sorted promptly. Events indicated in III, corresponding to total monocytes, were selected from SSC vs. LRP1 plot. Percentages of gated cells: 9.1%. Events in (III) were analyzed in SSC-W vs. SSC-H plot to exclude cellular aggregations obtaining percentages of gated cells approx. 95.4% (data not shown). The cell sorting was performed with a BD FACSAriaTMIIu cell sorter calibrated with BD Cytometer Setup Tracking Beads (No. 641319, BD Biosciences) and Accudrop Beads

Supplementary Figures

(No. 345248, BD Biosciences). BD FACSDivaTM software (version 6.1.2) was used to acquire and analyze the data.

Supplementary Figures

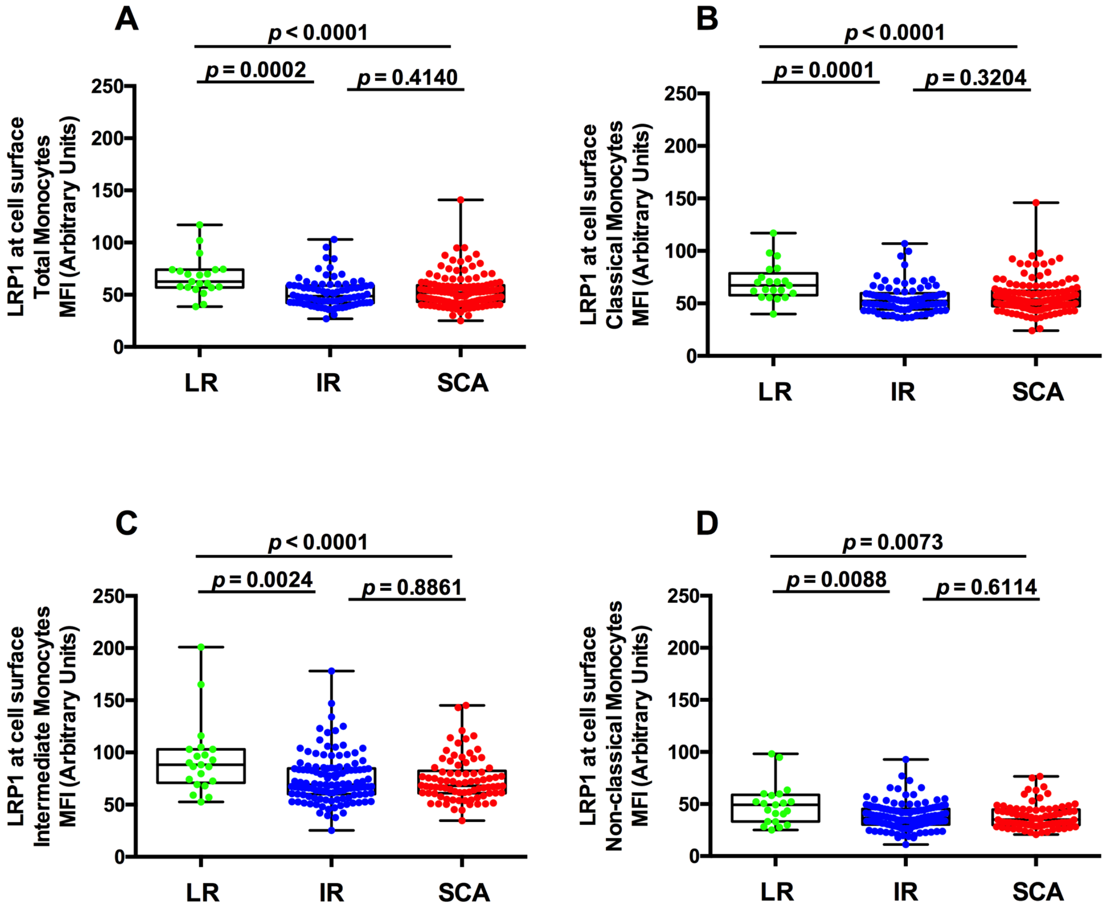

Supplementary Figure S2. LRP1 expression at cell surface in total monocytes and monocyte subsets. The expression level of LRP1 at cell surface was measured by flow cytometry assays in total monocytes (A), classical (B), intermediate (C) and non-classical (D) monocytes from peripheral blood extracted of individuals with low risk (LR) of CVD (green symbols; n=21), intermediate risk (IR) of CVD (blue symbols; n=124) and subclinical atherosclerosis (SCA) (red symbols; n=82). Results are shown as box plot representing the median value and interquartile range (IQR) of MIF (mean intensity of fluorescence). Maximal and minimal values of MFI for each study group are indicated (bar lines). Parameters were log-transformed to achieve normal distribution and to apply statistical parametric analysis, but in these table the original data were used. *p*-values are shown of SCA group vs. LR group. Differences were considered statistically significant when $p < 0.05$.

Supplementary Figures

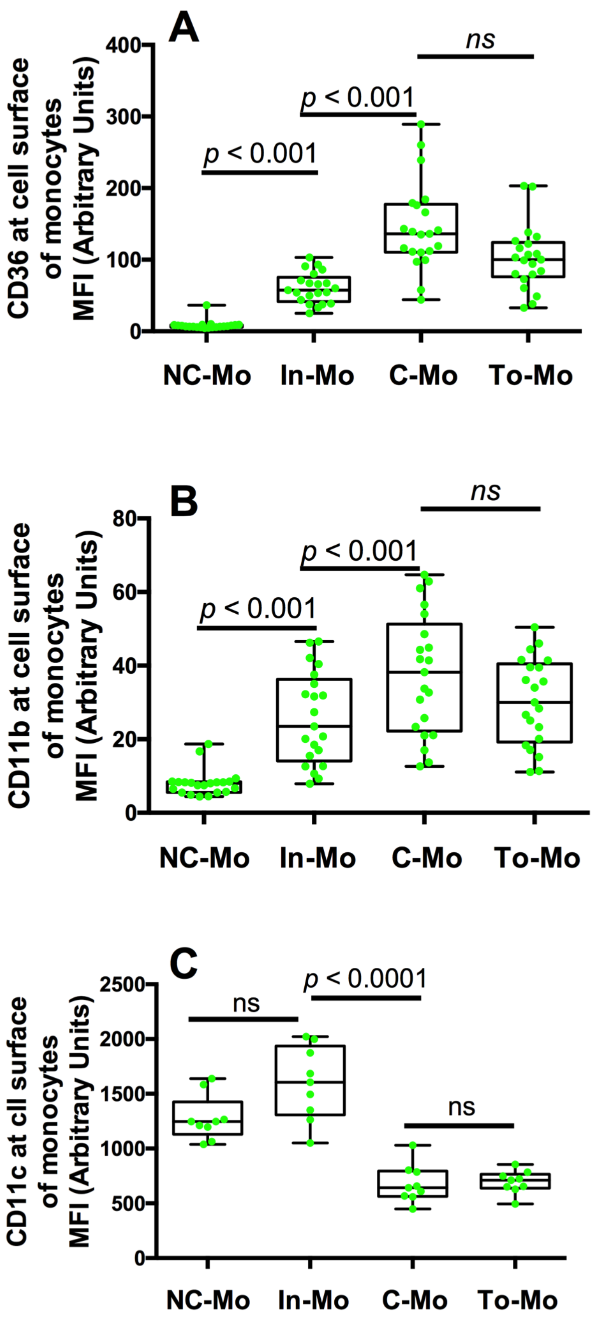

Supplementary Figures S3. CD36, CD11b and CD11c are differentially expressed at cell surface in total monocytes and monocyte subsets. These markers: CD36 (A), CD11b (B), and CD11c (C), were analysed by flow cytometry in non-classical monocytes (NC-Mo), intermediate monocytes (In-Mo), classical monocytes (C-Mo), and total monocytes (T-Mo) from peripheral blood of low risk (LR) individuals (green symbols). In A and B, twenty-one individuals with LR were included for these analyses, whereas for CD11c only eight individuals were analysed (Study I). Results are shown as box plot representing the median value and interquartile range (IQR) of MIF (mean intensity of fluorescence) for each marker and monocyte subset. Maximal and minimal values of MIF

are indicated (bar lines). p-values are shown to compare the marker expression between monocyte subsets and total monocytes and differences were considered statistically significant when $p < 0.05$. ns: non-significant result.

Supplementary Figures

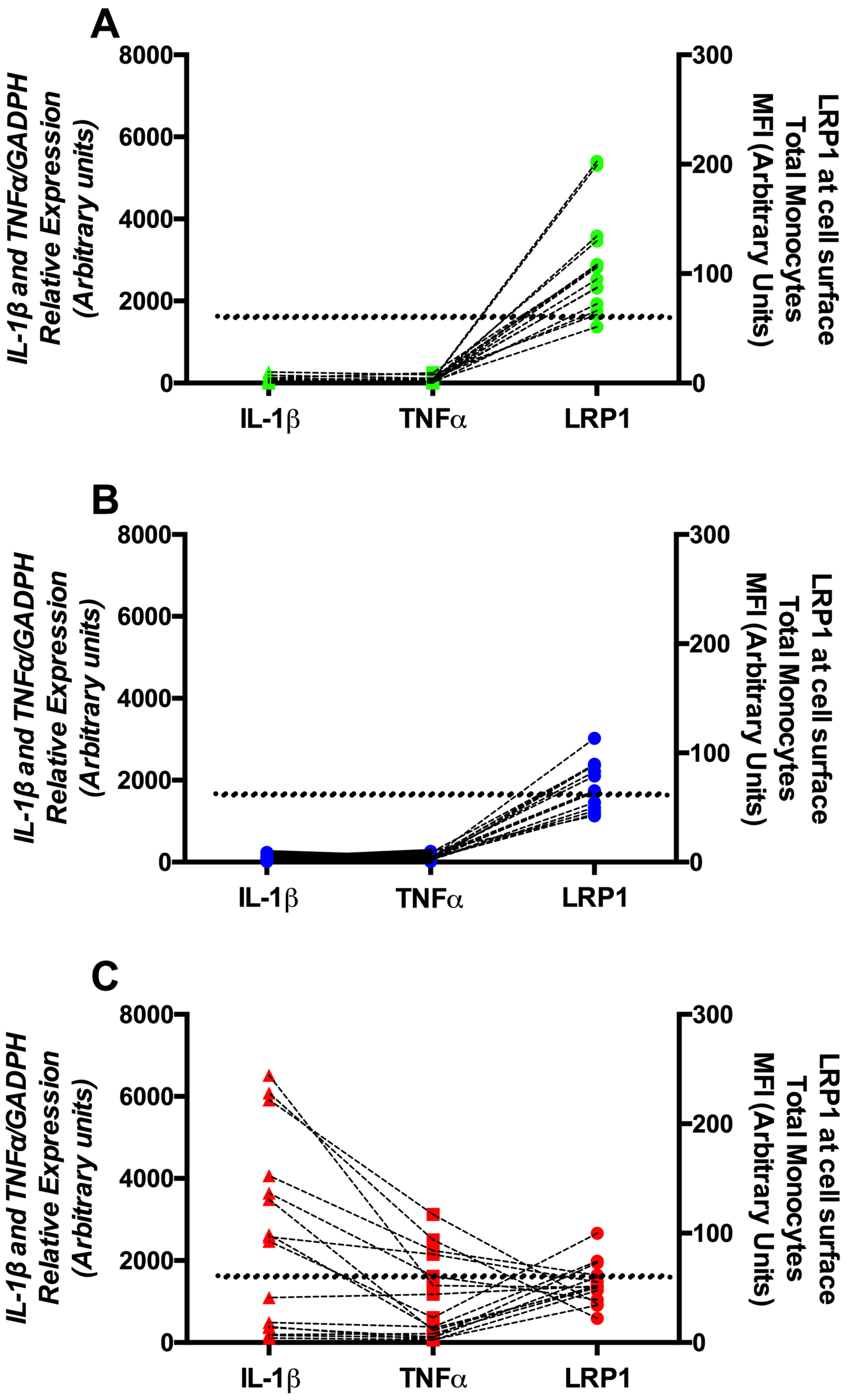

Supplementary Figures

Supplementary Figure S4. Comparative analysis of SCA individuals showed that decreased LRP1 expression in total monocytes mainly were associated with high levels of *TNF-α* and *IL-1β*. Levels of *TNF-α* and *IL-1β* mRNA in FACS-isolated total monocytes measured by qPCR (left y-axis) and LRP1 expression at cell surface in total monocytes measured by flow cytometry (right y-axis) in each individual with LR (**A**; green symbol; n= 16), IR (**B**; blue symbol; n= 16) and SCA (**C**; red symbol; n=16). In all both panels the dotted line is the cut-off value of MFI= 76 for LRP1 expression in total monocytes measured by flow cytometry in LR group and it represents the low limit of interquartile range (IQR) indicated in Figure 1B.
