# Supplementary material for: Decreased low-density lipoprotein receptor-related protein 1 expression in pro-inflammatory monocytes is associated with subclinical atherosclerosis
Source: Front Cardiovasc Med. 2022 Jul 26;9:949778. doi: 10.3389/fcvm.2022.949778 (PMC9360420; doi:10.3389/fcvm.2022.949778)
Supplement: Supplementary file 3 [file Data_Sheet_3.PDF]

Supplementary Table S1: Antigen-specific fluorochrome-labelled monoclonal antibodies used for flow cytometry assays.

Antigen	Antobody	Reporter	Manufacture	Clone	Isotype	Dilution	Cat#
**LRP1**	Anti-LRP1-α	PE	AbD Serotec	MCA19 65	Mouse IgG1	1/100	MCA1965P E
**CD14**	Anti-CD14	FITC	BD Biosciences	MφP9	Mouse IgG2b	1/50	561708
**CD16**	Anti-CD16	APC-Cy7	BD Biosciences	3G8	Mouse IgG1	1/50	557758
**CD45**	Anti-CD45	PC5	Beckam Coulter	J.33	Mouse IgG1	1/50	IM2653
**CD36**	Anti-CD36	PerCP-Cy5.5	BD Biosciences	CB38	Mouse IgM	1/100	561536
**CD11b**	Anti-CD11b	PerCP-Cy5.5	BD Biosciences	-------	Rat IgG2b	1/100	561114
**CD11c**	Anti-CD11c	PerCP-Cy5.5	BD Pharmingen	B-ly6	Mouse IgG1	1/100	565227

FITC, Fluorescein isothiocyanate; APC-Cy7, Allophycocyanin dye coupled with Cyanine 7; PE, R-phycoerythrin; PC5, phycoerythrin dye coupled with Cyanine 5; PerCP-Cy5.5, Peridinin-chlorophyll-protein Complex: CY5.5 Conjugate. AbD Serotec from Bio-Rad Laboratories, Inc. (Oxford, UK), BD Biosciences/Pharmingen (San Jose, CA); Beckam Coulter (Krefeld, Germany).

Supplementary Table S2: Specific primers for quantitative RT-PCR.

Gen	**NM/ID**	**Sequence: sense primer (F) anti-sense primer (R)**
LRP1	002332.2/4035	F 5´ -CTATGCACGCCCCTAAGACTT- 3´<br>R 5´-CATCGCTGGGCCTTACTCT- 3´
TNF-α	000594.3/7124	F 5´-TGCACTTTGGAGTGATCGGC -3´<br>*R 5´-GCTTGAGGGTTTGCTACAACA -3´*
IL-1β	000576.2/3553	F 5´-ATGATGGCTTATTACAGTGGCAA 3´<br>R 5´-GTCGGAGATTCGTAGCTGGA -3´
CCL2	002982.3/6247	F 5´-CTTCATTCCCCAAGGGCTC -3´<br>R 5´-GGTTTGCTTGTCCAGGTGGT -3´
CCR2	001123041.2/729230	F 5´-GGGATGACTCACTGCTGCAT -3´<br>R 5´-GGAGTGGGGCAATCCTACAG -3´
GAPDH	001256799.2/2597	F 5´-CTCCGGGTGATGCTTTTCCT 3´<br>R 5´-TGAAGGGGTCATTGATGGCA -3´

Supplementary Table S3: Correlation analysis between LRP1 expression at cell surface of total monocytes and CVD risk parameters

Parameters	Total Individuals (n=227)	Low risk group (LR) (n=21)	Intermediate group (IR) (n=124)	Subclinical atherosclerosis group (SCA) (n=82)
**BMI, kg/m^2**	***r*= -0,0835** ***p*= 0.2102**	***r*=** -0,0374 ***p*=** 0.8721	***r*=** 0,0713 ***p*=** 0.4314	***r*=** -0,1341 ***p*=** 0.2296
**Total Cholesterol, mg/dl**	***r*= -0.1718** ***p*= 0.0095**	***r*=** 0,1678 ***p*=** 0.4671	***r*=** -0,1457 ***p*=** 0.1064	***r*=** -0,0685 ***p*=** 0.5411
**Triglycerides, mg/dl**	***r*=** -0,1290 ***p*=** 0.0522	***r*=** 0,0327 ***p*=** 0.8880	***r*=** -0,0717 ***p*=** 0.4288	***r*=** -0,1087 ***p*=** 0.3312
**LDLc, mg/dl**	***r*=** -0,1235 ***p*=** 0.0632	***r*=** 0,3426 ***p*=** 0.1284	***r*=** -0,0302 ***p*=** 0.7395	***r*=** -0,0905 ***p*=** 0.4190
**Non-HDLc, mg/dl**	***r*= -0,1611** ***p*= 0.0151**	***r*=** 0,2796 ***p*=** 0.2197	***r*=** -0,0630 ***p*=** 0.4871	***r*=** -0,1043 ***p*=** 0.3511

LRP1 levels were log-transformed to achieve normal distribution and to apply the Pearson correlation analysis. Other parameters showed normal distribution. **BMI**, body mass index; **LDLc**, low density lipoprotein-cholesterol; **non-HDLc**, non-high density lipoprotein-cholesterol. Significant value, $p<0.05$. **Bold font** indicates parameter with statistical significance.

Supplementary Table S4: CD36, CD11b and CD11c expression at cell surface in total monocytes and monocyte subsets in LR, IR and SCA groups (Study I)

Monocyte marker MFI, arbitrary units	Without Subclinical Atherosclerosis		Subclinical Atherosclerosis (SCA) (n=82)	*p*-value* *Binary comparison***
	Low Risk Group (LR) (n=21)	Intermediate Risk Group (IR) (n=124)		
**CD36-Total Monocytes Median [IQR]**	100.0 [76.2-124.0]	91.6 [68.6-117.0]	86.6 [60.2-117.0]	0.4077 ***a***, *ns* ***b***, *ns* ***c***, *ns*
**CD36-Classical Monocytes Median [IQR]**	136.0 [110.5-177.5]	124.0 [91.8-169.8]	124.0 [87.4-162.0]	0.6453 ***a***, *ns* ***b***, *ns* ***c***, *ns*
**CD36-Intermediate Monocytes Median [IQR]**	57.6 [41.4-75.6]	52.6 [37.7-71.6]	51.6 [38.4-75.8]	0.7196 ***a***, *ns* ***b***, *ns* ***c***, *ns*
**CD36-Non-classical Monocytes Median [IQR]**	6.8 [5.9-8.8]	8.4 [6.7-10.4]	8.0 [7.0-9.9]	0.0934 ***a***, *ns* ***b***, *ns* ***c***, *ns*
**CD11b-Total Monocytes Median [IQR]**	30.0 [19.2-40.5]	23.7 [15.6-31.9]	25.3 [17.7-33.5]	0.1380 ***a***, *ns* ***b***, *ns* ***c***, *ns*
**CD11b-Classical Monocytes Median [IQR]**	38.2 [22.2-51.3]	28.5 [20.2-39.7]	30.3 [23.0-43.0]	0.2010 ***a***, *ns* ***b***, *ns* ***c***, *ns*
**CD11b-Intermediate Monocytes Median [IQR]**	23.5 [14.1-36.3]	18.2 [11.0-25.8]	21.8 [13.3-32.2]	0.0576 ***a***, *ns* ***b***, *ns* ***c***, *ns*
**CD11b-Non-classical Monocytes Median [IQR]**	8.0 [5.6-8.4]	6.6 [5.6-8.8]	7.4 [5.6-9.1]	0.3314 ***a***, *ns* ***b***, *ns* ***c***, *ns*
**CD11c-Total Monocytes Median [IQR]**	711 [639-766]	597 [419-705]	535 [450-656]	0.2065 ***a***, *ns* ***b***, *ns* ***c***, *ns*
**CD11c-Classical Monocytes Median [IQR]**	643 [563-795]	561 [410-700]	533 [456-664]	0.1558 ***a***, *ns* ***b***, *ns* ***c***, *ns*
**CD11c-Intermediate Monocytes Median [IQR]**	1606 [1307-1937]	1460 [1263-1665]	1468 [1288-1670]	0.5964 ***a***, *ns* ***b***, *ns* ***c***, *ns*
**CD11c-Non-classical Monocytes Median [IQR]**	1246 [1130-1425]	1147 [772-1365]	1020 [849-1224]	0.0920 ***a***, *ns* ***b***, *ns* ***c***, *ns*

Values are median (IQR, interquartile range). Parameters were log-transformed to achieve normal distribution and to apply statistical parametric analysis, but in these table the original data were used. * Ordinary one-way ANOVA; *ns*, non-significant. ***a***, ***b***, and ***c***: umpired *t*-test for mean values; ***a***, IR vs LR; ***b***, SCA vs LR; and ***c***, SCA vs IR. Significant value, $p<0.05$. **MFI**, mean fluorescence intensity.

Supplementary Table S5: Clinical and biochemical parameters (Study II).

Parameters	Without Subclinical Atherosclerosis		Subclinical Atherosclerosis Group (SCA) (n=16)	*p*-value* *Binary comparison***
	Low Risk Group (LR) (n=16)	Intermediate Risk Group (IR) (n=16)		
**Male (%)**	6 (37.5)	7 (43.7)	10 (62.5)	0.0082##
**Ages, years# Median [IQR]**	38.5 [25.2 – 42.0]	52.0 [50.0 – 54.7]	50.0 [33.2 – 55.7]	0.0002 ***a, b***
**BMI, kg/m2 Mean ± SD**	22.8 ± 2.5	26.4 ± 3.5	26.7 ± 3.0	0.0010 ***a, b***
**SBP, mmHg Mean ± SD**	107 ± 10	115 ± 13	113 ± 10	*ns*
**DBP, mmHg Mean ± SD**	73 ± 7	79 ± 7	79 ± 5	*ns*
**Total Cholesterol, mg/dl Mean ± SD**	158 ± 28	203 ± 73	200 ± 48	0.0353 ***a, b***
**LDLc, mg/dl Mean ± SD**	84 ± 19	98 ± 39	117 ± 37	0.0251 ***b***
**HDLc, mg/dl Mean ± SD**	61 ± 11	58± 21	46 ± 17	0.0442 ***b***
**Triglycerides, mg/dl Mean ± SD**	65 ± 21	207 ± 408	145 ± 84	*ns*
**Non-HDLc, mg/dl Mean ± SD**	97 ± 23	145 ± 77	154 ± 47	0.0094 ***a, b***
**Glucose, mg/dl Mean ± SD**	88 ± 5	97 ± 7	100 ± 8	<0.0001 ***a, b***
**Creatinine, mg/dl Mean ± SD**	0.8 ± 0.2	0.8 ± 0.2	0.8 ± 0.1	*ns*
**hs-CRP, mg/dl# Median [IQR]**	0.08 [0.04 – 0.15]	0.08 [0.04 – 0.14]	0.07 [0.04 – 0.16]	*ns*
**White cell count x 10^9/L# Median [IQR]**	7.3 [5.8 – 7.8]	6.5 [5.9 – 7.7]	7.0 [5.5 – 8.0]	*ns*
**Classical Monocytes, %# Median [IQR]**	76 [74 – 80]	71 [65 – 75]	74 [69 – 78]	*ns*
**Non-Classical Monocytes, %# Median [IQR]**	8.6 [6.5–11.3]	11.0 [7.5–18.7]	10.6 [7.9–15.0]	*ns*
**Intermediate Monocytes, %# Median [IQR]**	6.3 [5.0-9.6]	6.5 [5.4-7.8]	6.8 [5.8-8.1]	*ns*
**CACS, Agatston Units**	0	0	>1.0 F: 1/6 M: 7/10	

Values are mean ± SD (standard deviation) for normal distribution, or median (IQR, interquartile range) for non-normal distribution. **BMI**, body mass index; **SBP**, systolic blood pressure; **DBP**, diastolic blood pressure; **LDLc**, low density lipoprotein-cholesterol; **HDLc**, high density lipoprotein-cholesterol; **non-HDLc**, non-high-density lipoprotein-cholesterol; **hs-CRP**, high sensitivity - C-reactive protein; **CACS**, coronary artery calcium score. **#**Parameters were log-transformed to achieve normal distribution and to apply statistical parametric analysis, but in these table the original data were used. ##Contingency analysis and Chi-square test. * Ordinary one-way ANOVA; *ns*, non-significant. ****a***, ***b***, and ***c***: umpired *t*-test for mean values; ***a***, IR vs LR; ***b***, SCA vs LR; and ***c***, SCA vs IR. Letters with significant value, *p*<0.05, are shown; **ns**, non-significant *p* value.

Supplementary Table S6: Correlation analysis between cell surface LRP1 expression and pro-inflammatory factor in total monocytes (Study II)

	LRP1 expression in total monocytes (MFI values) vs.				*LRP1/GADPH* mRNA levels (relative expression) vs.			
	TNFα/GADPH ***(Relative Expression)***	***IL-1β/GADPH*** ***(Relative Expression)***	***CCL2/GADPH*** ***(Relative Expression)***	***CCR2/GADPH*** ***(Relative Expression)***	***TNFα/GADPH*** ***(Relative Expression)***	***IL-1β/GADPH*** ***(Relative Expression)***	***CCL2/GADPH*** ***(Relative Expression)***	***CCR2/GADPH*** ***(Relative Expression)***
r	**-0.376**	**-0.387**	-0.257	-0.173	0.009	-0.008	-0.0879	-0.2561
p	**0.008**	**0.007**	0.077	0.241	0.949	0.959	0.5527	0.079

All parameters were log-transformed to achieve normal distribution and to apply the Pearson correlation analysis. Significant value, $p<0.05$. **Bold font** indicates parameter with statistical significance. **MFI**, mean fluorescence intensity; (n= 48)
